# Viral communities in the parasite *Varroa destructor* and in colonies of their honey bee host (*Apis mellifera*) in New Zealand

**DOI:** 10.1038/s41598-022-12888-w

**Published:** 2022-05-25

**Authors:** Philip J. Lester, Antoine Felden, James W. Baty, Mariana Bulgarella, John Haywood, Ashley N. Mortensen, Emily J. Remnant, Zoe E. Smeele

**Affiliations:** 1 Centre for Biodiversity and Restoration Ecology, School of Biological Sciences, Victoria University of Wellington, PO Box 600, Wellington, 6012 New Zealand; 2 School of Mathematics and Statistics, Victoria University of Wellington, PO Box 600, Wellington, 6012 New Zealand; 3 The New Zealand Institute for Plant and Food Research Limited, Private Bag 3230, Waikato Mail Centre, Hamilton, 3240 New Zealand; 4 Behaviour, Ecology and Evolution Laboratory, School of Life and Environmental Sciences, University of Sydney, Science Road, Sydney, NSW 2006 Australia

**Keywords:** Virus-host interactions, Ecological epidemiology, Entomology

## Abstract

The parasitic mite *Varroa destructor* is a leading cause of mortality for Western honey bee (*Apis mellifera*) colonies around the globe. We sought to confirm the presence and likely introduction of only one *V. destructor* haplotype in New Zealand, and describe the viral community within both *V. destructor* mites and the bees that they parasitise. A 1232 bp fragment from mitochondrial gene regions suggests the likely introduction of only one *V. destructor* haplotype to New Zealand. Seventeen viruses were found in bees. The most prevalent and abundant was the *Deformed wing virus A* (DWV-A) strain, which explained 95.0% of the variation in the viral community of bees. *Black queen cell virus*, *Sacbrood virus*, and *Varroa destructor virus 2* (VDV-2) played secondary roles. DWV-B and the *Israeli acute paralysis virus* appeared absent from New Zealand. Ten viruses were observed in *V. destructor*, with > 99.9% of viral reads from DWV-A and VDV-2. Substantially more variation in viral loads was observed in bees compared to mites. Where high levels of VDV-2 occurred in mites, reduced DWV-A occurred in both the mites and the bees co-occurring within the same hive. Where there were high loads of DWV-A in mites, there were typically high viral loads in bees.

## Introduction

**Figure 1:**
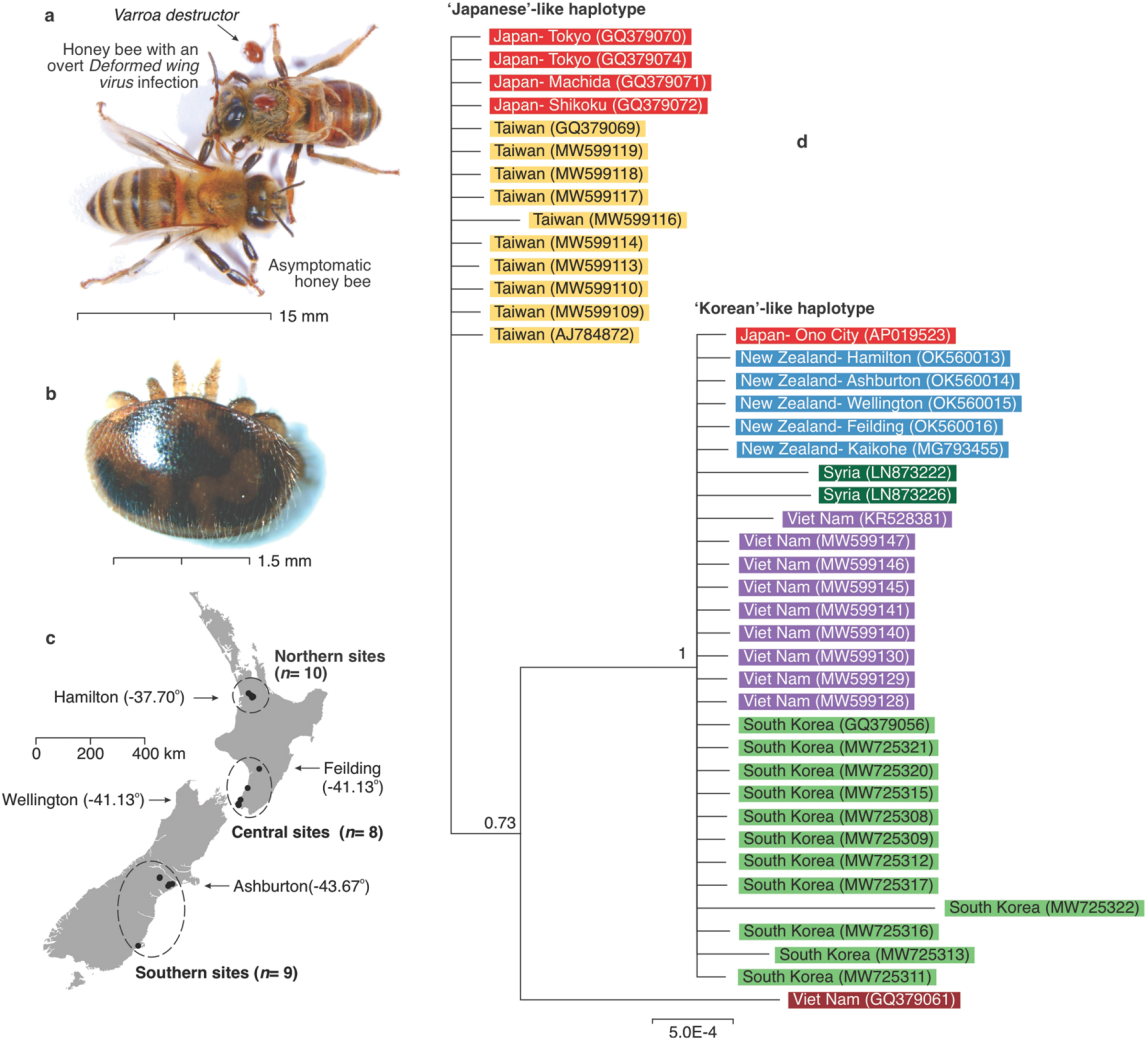
(**a**) A Western honey bee (*Apis mellifera*) showing an overt *Deformed wing virus* (DWV) infection, with *V. destructor* mites, and another bee asymptomatic for this virus. (**b**) *Varroa destructor* mites were sampled alive and typically moved quickly when disturbed. This mite was sampled from the Ashburton location. It had slow movement and unusually dark staining, which might indicate it was old, although some pathogens can also alter *V. destructor* physiology and behaviour^37^. (**c**) Sample sites in New Zealand showing the location of hives from which both adult honey bees and *V. destructor* mites were sampled. The sites are grouped into three regions for the statistical analysis. The arrows with latitude south coordinates show the location of the five *V. destructor* samples used in the phylogenetic tree. An additional sample of nine mites showed no genetic variation. (**d**) A Bayesian phylogeny showing the grouping of *V. destructor* mitochondrial haplotypes for 862 bp of the cytochrome c oxidase I (MT-CO1) gene. The samples from New Zealand (coloured blue) clustered within the Korean-like haplotype. The number between brackets is the GenBank accession number. Support for clades correspond to posterior probabilities. Photographs by Phil Lester.

The parasitic mite *Varroa destructor* is currently considered to be the most serious threat to Western honey bee (*Apis mellifera*) colonies^1–3^ (Fig. 1a,b). *Varroa destructor* evolved on the Eastern honey bee, *Apis cerana*, but was introduced to Western honey bees during the first half of the last century and has since dispersed to nearly every country in the world^4^. The mite primarily feeds on the fat body of bees^5^, thereby suppressing the immune response of parasitised bees or bee colonies^1,6,7^. Critical to the impact of the mite are the viruses that it vectors or exacerbates. Viral infections only became a widespread and serious health issue for honey bees after *V. destructor* infestation^8,9^. *Varroa destructor* are known to host a diverse viral community^10–13^. Of these viruses the *Deformed wing virus* (DWV) is considered to be the key pathogen associated with honey bee over-wintering mortality^14^. Direct DWV inoculation mediated by *V. destructor* can change the viral community within the bee host^15^. There is a ‘swarm’ of DWV variants^16^ and many strains of DWV that can occur in bees without *V. destructor*, but after the introduction of the parasite one or two strains predominate^17,18^. One emerging DWV strain is known as DWV-B (previously referred to as *Varroa destructor virus* 1 or VDV-1). DWV-B appears to be more virulent than the originally described variant DWV-A^19^. The DWV-B genotype seems to be replacing the DWV-A strain in several countries^20–22^ and has been linked to overwintering honey bee losses^23^.

*Varroa destructor* was first identified in New Zealand in 2000, although it may have been present for 3–5 years prior to this time^24^. A previous analysis of a 929 bp fragment of the cytochrome c oxidase I (MT-CO1) gene region of six individual *V. destructor* from 2005 indicated a single introduction, consisting of one haplotype of the ‘Korean’ strain^25^. It seems likely that the mite was introduced via the illegal importation of a honey bee queen or queens accompanied by attending workers^24^. DWV was probably first introduced to New Zealand with these bees and *V. destructor*^26^. Mites are now present in at least 65–70% of New Zealand hives and apiaries sampled in autumn^26^. Mondet et al.^27^ previously examined viruses in both *V. destructor* and honey bees in New Zealand. They specifically examined the infection prevalence and viral titres of seven viruses. These viruses, ordered from most to least prevalent in honey bees (when in the presence of mites) were the *Black queen cell virus* (BQCV), DWV, *Sacbrood virus* (SBV), *Kashmir bee virus* (KBV), and the *Chronic bee paralysis virus* (CBPV). The *Acute bee paralysis virus* (ABPV) and the *Israeli acute bee paralysis virus* (IAPV) were not observed in New Zealand mites or bees^27^. Other studies have since confirmed the absence of IAPV from New Zealand^28^ and a high prevalence of DWV and BQCV in bees^26^. In *V. destructor*, Mondet et al.^27^ found the prevalence of viruses was DWV, BQCV, KBV, SBV, and CBPV, from most to least prevalent, respectively. Non-random patterns of viral communities were observed in mite and bee samples, with the DWV titres in bees and *V. destructor* correlated. Their work also suggested that viral communities changed either with increasing time since *V. destructor* arrival, or perhaps in a latitudinal cline. Higher DWV titres were observed in northern apiaries where *V. destructor* had been established the longest^27^. DWV and many other ‘honey bee’ viruses are now seen in a wide variety of arthropods in New Zealand^29–33^.

RNA-Seq technology has been used to enhance viral discovery and quantification for both honey bees and *V. destructor*^9,22^. For example, Levin et al.^34^ used RNA-Seq to examine viruses in honey bees and mites in Israel. Their analysis found eight viral species in bees. In *V. destructor* from the same hives*,* 22 viral species were found including two that had not been previously described. DWV-A and DWV-B (previously known as VDV-1) dominated both communities as assessed by read abundance. The viral species newly discovered using RNA-Seq were only found in the mites but not in bees they parasitised^34^. Work on Middle East and African honey bees has also shown a high viral diversity in *V. destructor* relative to their bee hosts. Other RNA-Seq studies from Asia have similarly identified new viruses prevalent in *V. destructor*, sometimes demonstrating a *V. destructor* virus to be present and replicating in associated honey bees^10,11^. DNA viruses have similarly been recently discovered in mites^12,22,35^, some of which they may passively acquire from feeding on bees^36^. At least 59 putative viral species have previously been identified from *V. destructor* (Supplementary Table S1). Two key conclusions can be drawn from these studies. Firstly, several newly discovered viruses have recently been described from *V. destructor* and bees, although their distribution, prevalence and effects on hosts are largely unknown. The viral communities in *V. destructor* may influence the life history of the bees they parasitise^6^ as well as that of the mite themselves^37^. Secondly, the abundance of several of these viral species in bees and mites may be correlated^10,27^, while other viruses appear unique to each animal.

Our goals in this study were to: (i) confirm the presence and likely introduction of only one *V. destructor* haplotype in New Zealand; (ii) assess and describe the over-wintering viral community within both *V. destructor* mites and the bees that they parasitise, in order to facilitate our understanding of how the viral community in mites might influence that of bees; and (iii) examine the viral landscape within New Zealand mites and honey bees for regional variation.

## Materials and methods

### Sample collection

We collected both *V. destructor* and bees from 27 colonies within New Zealand during autumn to winter (May–August) 2021. It is over the autumn–winter period that honey bee colony losses have been linked with increased prevalence of viral infections, particularly with DWV^23^. We opportunistically sampled hives from a mixture of hobbyist, commercial and research hives throughout New Zealand, which were broadly categorised into regions or sites from the upper North Island, lower North Island and South Island (Fig. 1c, Supplementary Table S2). Samples were taken directly from hives by the authors, or bees and mites were sent by beekeepers to Victoria University of Wellington. *Varroa destructor* is listed as an ‘Unwanted Organism’ in New Zealand under the Biosecurity Act 1993, so permission to send and handle this pest was obtained from the Ministry for Primary Industries under sections 52 and 53 of the Biosecurity Act. A diversity of mite management approaches had been used on the hives in the previous seasons ranging from no mite treatments, chemical pesticides including amitraz and flumethrin, and organic approaches with oxalic acid. The relative density of mites may have fluctuated considerably leading up to the point of collection and beekeepers who sent samples used a variety of approaches in their collection. Hence, we did not record the mite control treatments used or the relative density of mites, instead focussing solely on collecting at least 10 mites and 10 bees from each hive. Pooled samples of 5–10 individuals have been used in previous pathogen surveys from honey bee hives^38–40^. From two samples, only 5 and 8 mites were able to be recovered. Live mite and bee samples were anesthetised using CO_2_ and placed directly into a − 80 ºC freezer or were snap-frozen in liquid nitrogen.

### Mitochondrial DNA haplogroup assignment

We first sought to confirm the presence and likely introduction of only one *V. destructor* haplotype in New Zealand. DNA was extracted from 13 individual mites, from different hives in three locations in New Zealand to determine their mitochondrial DNA haplogroup (Fig. 1d, Supplementary Table S3). DNA was extracted using GENEzol DNA reagent plant (Geneaid, Taiwan). Three partial mitochondrial genes, cytochrome c oxidase I (MT-CO1), cytochrome c oxidase III (MT-CO3) and ATP synthase membrane subunit 6 (MT-ATP6) were amplified using the primers developed by Navajas et al.^41^. We performed PCR in 25 μL reactions, consisting of 2 μL mite DNA, 12.5 μL MyTaq Red Mix (Meridian Bioscience, USA), 1 μM forward primer, 1 μM reverse primer, and nuclease-free water. Cycling conditions followed Navajas et al.^41^ but with 40 cycles. A non-template control was included for each target. PCR products were visualised on 2% agarose gels and were then treated with Exo-CIP Rapid PCR Cleanup Kit (New England BioLabs, USA) prior to sequencing. Samples were sequenced on an ABI3730 DNA Analyzer at Massey Genome Service (Palmerston North, New Zealand). Sequence base-calls were checked by eye using Geneious^42^. We aligned sequences from different mites with the Geneious alignment algorithm, using global alignment with free ends and gaps and a cost matrix equal to 93% similarity.

To compare *V. destructor* mites found in New Zealand with those in other countries, we downloaded *V. destructor* sequences from GenBank that overlapped with the gene portions that we sequenced. Sequences were aligned as explained above. Clade probabilities were obtained from the posterior distribution using the MrBayes v.3.2.6 plug-in^43^ for Geneious. Bayesian analyses were replicated twice, each with four Markov chains of 1 million generations. Trees were sampled every 2,500 generations, of which the first 150,000 generations were discarded as burn-in. Sequences have been archived in GenBank (accession numbers in Supplementary Table S3).

### Viral communities in *V. destructor* and bees using RNA-Seq

For each bee sample, 10 adult bees from the same hive were pooled into a 7 mL reinforced tube with three 3.2 mm stainless steel beads (Next Advance Inc., USA). The samples were homogenised for 3 cycles of 30 s each at 7500 rpm in a Precellys Evolution homogeniser (Bertin Instruments, France) with the dry ice on the top compartment to keep the samples cold. We added ~ 0.5 g of the bee homogenate to a centrifuge tube containing 600 µL of cold TRIzol reagent (Life Technologies, USA). The remainder of the extraction procedure followed the Direct-Zol RNA MiniPrep kit protocol (Zymo Research, USA). The concentration and purity of RNA samples were determined using a NanoPhotometer NP80 (Implen, Germany), and samples stored immediately at − 80 °C. For each pooled mite sample, RNA was extracted as described above using the Direct-Zol RNA MicroPrep kit (Zymo Research, USA).

Bee and mite samples were shipped dried in Gentegra RNA tubes (Gentegra, USA) via Custom Science (Auckland, New Zealand) for 150 base pair (bp) paired-end sequencing with NovaSeq 6000 (Illumina, USA). Raw reads were processed using *Trimmomatic* 0.39 to remove bases with a mean Phred score < 20 over a sliding window of 4 bp as well as reads shorter than 25 bp^44^. Adapters were trimmed off using the adapter sequences which were provided by the sequencing company, i.e. universal Illumina adapters P7 and P5. Clean read quality was checked using *FASTQC* 0.11.7 (Babraham Institute, UK). We aligned bee and *V. destructor* clean reads using *HISAT2* 2.1.0^45^ onto the honey bee^46^ and *V. destructor*^47^ genomes, respectively. Files containing unmapped reads generated by *HISAT2* were used as input for de novo transcriptome assembly with *Trinity* 2.13.2^48^. We ran separate assemblies for bee and *V. destructor*. In order to identify viral transcripts, we used *DIAMOND*^49^ to align the resulting assembled transcripts on the viral protein NCBI database^50^ downloaded on 06/10/2021. Significant alignments with an e-value < 1 × 10^–5^ were then re-aligned to the non-redundant protein database using *DIAMOND* in order to remove false-positives and include viral transcripts only deposited in the NCBI non-redundant protein database^50^ downloaded on 04/11/2021. The outputs from *DIAMOND* were processed using a custom *R* script that selected for the hits with the best bit score, e-value and percent identity, in that order. Final *DIAMOND* hits were verified using manual *BLASTn*^51^. We obtained viral transcript abundance expressed as transcripts per million (TPM) using *Salmon* implemented within *Trinity* (align_and_estimate_abundance.pl script)^52^. We further normalised TPM values to the number of host reads taken from the *HISAT2* step statistics (i.e. reads that aligned to the *V. destructor* or honey bee reference genomes). We only retained viruses known to infect arthropods.

All analysis were performed within the R statistical environment^53^. We used two separate principal components analyses (PCA) to represent the unscaled variation in the viral loads from bees and from *V. destructor*, as in our previous work^32^. From both PCAs, we retained only the viruses associated with the first two principal components, which accounted for almost all (> 99.9%) of the total variance in each case. We then calculated pairwise Spearman correlations for the viruses retained from each PCA, examining viral loads between and within bees and *V. destructor*. In order to investigate the associations between DWV in bees and VDV-2 plus DWV in *V. destructor*, we also investigated pairwise linear regressions. We tested for differences in viral communities between regions in bees and *V. destructor*, using PERMANOVA from the *adonis* function in the *vegan* package with both the Bray–Curtis (abundance) and Jaccard (presence/absence) methods^54^.

### PCR and replication assays for selected RNA viruses

We further interrogated our samples for the presence of DWV-A, DWV-B (previously described as VDV-1), BQCV, KBV, IAPV, VDV-2, VDV-3 and VDV-5, using reverse-transcription (RT)-PCR. Sample RNA was combined resulting in one bee RNA pool and one *V. destructor* RNA pool for each region, which were then used to generate cDNA using Quanta qScript cDNA SuperMix (Quantabio, USA) following the manufacturer’s instructions. Equal amounts of cDNA from each region for *V. destructor* and bee samples were then pooled to create one *V. destructor* cDNA master mix and one bee cDNA master mix that was used for PCR assays. The samples were pooled specifically because we wanted only to provide evidence on whether viral replication was occurring in mites or in bees (rather than determining, for example, how frequently replication was occurring in each species). PCR was then carried out using primer sets described in Supplementary Table S4. PCR reactions consisted of 7.5 uL MyTaq Red Mix, 1 µL of 10 µM forward primer, 1 µL of 10 µM reverse primer, 1 µL cDNA, made to 15 µL with nuclease-free water. PCR cycling conditions were: 95 °C for 5 min; 35 cycles of 95 °C for 15 s, 58 °C for 30 s, 72 °C for 60 s, with a final extension at 72 °C for 5 min and a hold step at 4 °C. PCR products were separated using 2% agarose gel electrophoresis and visualized using SYBR Safe DNA gel stain (Invitrogen/ThermoFisher Scientific, USA). To prepare PCR products for Sanger sequencing, samples were digested with ExoSAP-IT PCR Product Cleanup Reagent (Applied Biosystems/ThermoFisher Scientific, USA). Samples were then sent to Massey Genome Service (Palmerston North, New Zealand) for sequencing. Geneious was used to analyze sequences^42^.

For positive-sense single-stranded RNA viruses, the presence of the negative-sense strands is indicative of viral replication. Strand-specific RT-PCR assays were used to detect the negative-sense viral strands for DWV-A, DWV-B, BQCV, KBV, IAPV, VDV-2, VDV-3 and VDV-5. Tagged-forward primers (Supplementary Table S4) were used to generate cDNA from the negative-strand using the Super-Script IV First-Strand Synthesis System (Invitrogen/ThermoFisher Scientific, USA). PCR was then conducted as above.

### DNA virus confirmation assays

We also examined our samples for the *Apis mellifera filamentous virus* (AmFV), which is a large double stranded DNA virus from honey bees. DNA was extracted from pools of 10 mites, and separately 10 bees, taken from hives within three regions: the upper North Island, lower North Island and South Island (*n* = 3, Fig. 1c, Supplementary Table S5). Briefly, each 10-mite or 10-bee sample was homogenized by bead-beating in microcentrifuge tubes containing 1 mL of GENEzol plant DNA reagent (Geneaid Biotech, Taiwan) and 5 µL of β-mercaptoethanol (Sigma Aldrich, USA). Samples were homogenised for 3 cycles of 15 s each at 6,000 rpm, followed by 1 cycle of 10 s at 9,900 rpm. We used a 24:1 chloroform–isoamyl alcohol mixture (BioUltra from Merck, USA) to isolate the nucleic acids, followed by isopropanol precipitation (BioReagent from Merck, USA), and a 70% ethanol purification step (VWR Chemicals, UK). Mite DNA was eluted in 25 µL and bee DNA in 100 µL of nuclease-free water.

We screened these pooled mite and bee samples respectively for AmFV via PCR. We used the primer sets designed by Cornman et al.^35^ to amplify two portions of the pathogen genome^35^: a ribonucleotide reductase small subunit gene and a thymidylate synthase gene. We amplified each locus in a 15 μL PCR containing 1 μL DNA, 1 μM forward primer, 1 μM reverse primer, water and 7.5 μL MyTaq Red Mix. Cycling conditions were: 1 min at 95 °C; 35 cycles of 15 s at 95 °C, 15 s at 54 °C, and 10 s at 72 °C; followed by a final extension of 5 min at 72 °C. We included a non-template control in each reaction. PCR products were visualised on a 2% agarose gel. We cleaned up the samples using the Monarch DNA Gel Extraction Kit (New England BioLabs, USA). The identities of the products were confirmed by Sanger sequencing (GenBank accession numbers in Table S5). Sequence base-calls were checked by eye using Geneious^42^. We used the *BLASTn* algorithm to search against the nucleotide database in NCBI GenBank. Gene identifications were assigned to genes on the database based on sequence identity > 97%. We aligned the sequences with the Geneious alignment algorithm.

## Results

### Mitochondrial DNA haplogroup assignment

Our first goal was to confirm the presence and likely introduction of only one *V. destructor* haplotype in New Zealand. We sequenced a total of 1232 bp from three mitochondrial gene regions of *V. destructor* taken from 13 spatially separated hives (Fig. 1c, Supplementary Table S3). There was no variation between any of these sequences (Fig. 1d). Further, our samples showed no variation from one 811 bp sequence from two New Zealand *V. destructor* samples that were deposited on GenBank sampled in 2016 from Kaikohe and Gisborne^55^. This single New Zealand haplotype is most similar to the Korean-like haplotype of *V. destructor*^56^. Because of the absence of any genetic variation within the New Zealand haplotypes, it seems likely that there has been only one successful *V. destructor* introduction and establishment here. We note, however, that it would be impossible to prove this conclusion without widespread and extensive sampling throughout the country. Mitochondrial DNA sequences of similar length from other countries show variation indicative of multiple haplotype introductions (Fig. 1d).

### Viral communities in bees and *V. destructor* using RNA-Seq

**Figure 2:**
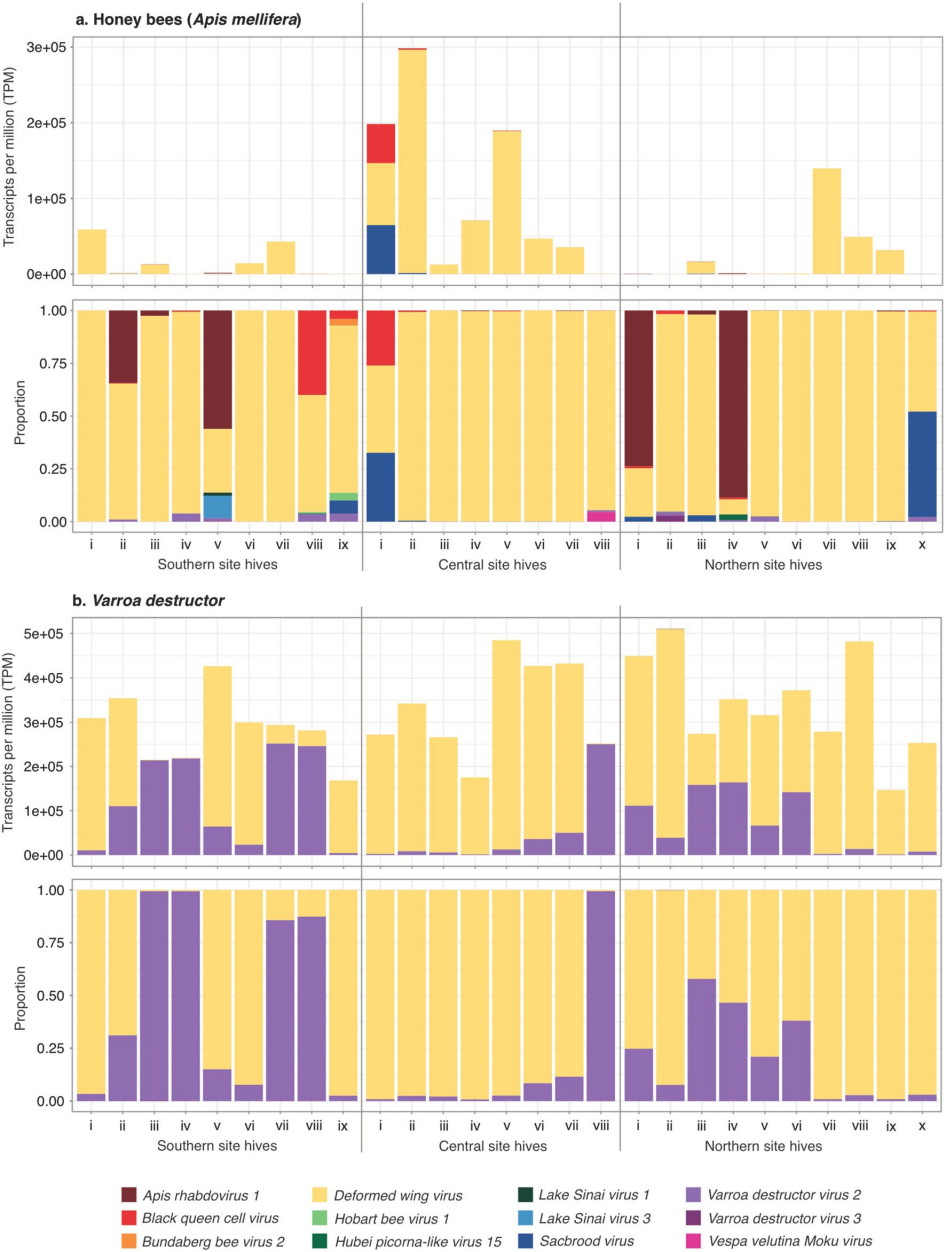
Viruses in honey bees and *V. destructor* mites. (**a**) DWV-A was the most abundant and prevalent in honey bees, occurring in all bee samples from all 27 hives that we sampled, although with substantial variation between samples. A total of 17 virus species were seen in these bee samples. (**b**) In comparison to the bees, the viral loads in mites showed much less variation. The viral community of *V. destructor* was dominated by DWV-A and VDV-2. Eight other viruses were observed in relatively low abundance from these mites.

We found strong evidence for 17 viruses in our honey bee samples (Table 1; Fig. 2a; Supplementary Table S6). Each individual hive contained multiple RNA viral species infections within the inhabiting bees. The median number of RNA viruses observed from a bee sample within a hive was 7 (range: 4–11 across all 27 hives). DWV-A was found in all 27 bee samples with viral loads representing between 7.1 and > 99.9% of the total viral load in our samples. We found SBV in 22 samples (< 0.01–49.7% of total viral loads), BQCV in 25 samples (< 0.01–39.9% of total viral loads), and VDV-2 in 26 samples accounting for < 0.01–3.9% of total viral loads. *Apis rhabdovirus 1* was present in 25 samples, typically in small numbers of transcripts but represented more than a third of total viral loads in four samples (overall < 0.01–88.6% of the total viral loads). *Apis rhabdovirus 2* was found in nine samples but at low levels (up to 0.02% of total viral loads). We observed *Vespa velutina Moku virus* in five samples (< 0.01–4.6% total viral loads), VDV-5 in four samples (< 0.01–2.9% of total viral loads) and *Hobart bee virus* in three samples (< 0.01–3.7% total viral loads). *Lake Sinai virus 1* was found in eight samples (< 0.01–1.5% of total viral loads), and *Lake Sinai virus 3* was only found in one sample, but in this sample accounted for 10.5% of the total viral load. Other less abundant viruses were only found at < 1% of total viral loads in samples where they were present. For example, KBV was found in 18 samples but only accounted for ≤ 0.7% of viral loads. *Aphid lethal paralysis virus* was only found in five North and Central region samples (< 0.01–0.4% of total viral loads). CBPV was found in three samples (0.01–0.3% of total viral loads). There was evidence for additional viruses present in our sequences, though we excluded these for the purpose of our analyses due to their low sequence identity or high e-value scores. In our PERMANOVA we found no significant differences in bee viral communities between regions, using either abundance data (*F* = 1.899, *R*^*2*^ = 0.137, *p* = 0.160) or presence/absence data (*F* = 0.696, *R*^*2*^ = 0.124, *p* = 0.154).

**Table 1:** Viruses observed in honey bees and mites from the RNA-Seq analysis. The counts are expressed as the average transcript abundance, expressed in transcripts per million (TPM). The viruses are ordered by the most to least common virus observed in honey bees. ‘S.E.’ is the standard error; ‘Infected’ is the percentage of the 27 hives from which the mite or bee samples had reads for each virus. ‘−’ indicates no virus reads were found in a sample. For the average, standard error, and range calculations we excluded the 0 values.

Virus	Honey bees				*Varroa destructor*			
	Average	S.E	Range	Infected (%)	Average	S.E	Range	Infected (%)
Deformed wing virus (DWV-A)	40,737	13,183	(76– 294,978)	100	238,459	27,545	(1265– 472,301)	100
Sacbrood virus (SBV)	3030	2937	(0.05– 64,693)	81	31	15	(0.07– 265)	74
Black queen cell virus (BQCV)	2188	2066	(0.04– 51,721)	93	12	7	(0.02– 75)	48
Lake Sinai virus 3 (LSV-3)	175	–	–	4	–	–	–	–
Apis rhabdovirus 1 (ARV-1)	150	54	(0.02– 941)	93	44	22	(0.42– 592)	100
Kashmir bee virus (KBV)	92	75	(0.03– 1333)	67	37	21	(0.30– 174)	33
Lake Sinai virus 1 (LSV-1)	34	23	(0.02– 185)	30	–	–	–	–
Drosophila subobscura Nora virus	14	–	–	4	–	–	–	–
Chronic bee paralysis virus (CBPV)	13	13	(0.04– 40)	11	–	–	–	–
Varroa destructor virus 2 (VDV-2)	11	2	(0.06– 30)	96	81,853	17,255	(1,295– 251,109)	100
Bundaberg bee virus 2	9	2	(7– 10)	7	3	2	(0.46– 6)	11
Varroa destructor virus 5 (VDV-5)	6	2	(4– 10)	15	124	29	(1– 551)	100
Hobart bee virus 1	5	4	(0.23– 12)	11	–	–	–	–
Vespa velutina Moku virus	4	3	(1– 16)	19	–	–	–	–
Aphid lethal paralysis virus (ALPV)	4	3	(1– 16)	19	–	–	–	–
Hubei picorna-like virus 15	4	3	(0.17– 28)	37	–	–	–	–
Apis rhabdovirus 2 (ARV-2)	1	0.46	(0.04– 4)	33	34	8	(1– 174)	100
Hubei picorna-like virus 22	–	–	–	–	9	7	(0.09– 37)	19

We found 10 viruses in *V. destructor*, including nine shared with honey bees. Each individual hive contained multiple RNA viral species infections within the inhabiting mites. The median number of RNA viruses observed from a *V. destructor* sample within a hive was 7 (range: 5–10 across all 27 hives). DWV-A and VDV-2 were found in all samples, accounting for 0.6–99.2% and 0.8–99.3% of total viral loads, respectively (Table 1; Fig. 2b; Supplementary Table S7). These two viruses accounted for > 99.9% of the observed average viral loads in these mites (Table 1). All the other viruses were observed at low viral loads, with each accounting for < 0.04% of the average number of viral loads observed. ARV-1, ARV-2 and VDV-5 were found in all samples, but only accounted for ≤ 0.3% of any sample total viral loads. SBV, BQCV and KBV were found in 20, 13 and nine samples, respectively. *Hubei picorna-like virus 22* was present in five samples (≤ 0.008% of total viral loads) and *Bundaberg bee virus 2* in three samples (≤ 0.002% of total viral loads). We found no significant differences in *V. destructor* viral communities between regions, using either abundance data (*F* = 1.257, *R*^*2*^ = 0.095, *p* = 0.254) or presence/absence data (*F* = 0.988, *R*^*2*^ = 0.076, *p* = 0.434).

We found no evidence in the RNA-Seq data for the viruses DWV-B, IAPV, and VDV-3. Only the DWV-A strain was detected.

### Principal component analyses and association between viruses

In bees, DWV-A was strongly associated with the first principal component (PC1_bees_, Supplementary Table S8), which explained 95.0% of the total variance in the dataset. The second principal component (PC2_bees_) was associated predominantly with SBV and BQCV. Together the first two principal components explained more than 99.99% of the total variance. In the *V. destructor* analysis, DWV and VDV-2 were effectively the only viruses associated with the first two principal components, which together explained 99.9% of the total variance (Supplementary Table S9).

**Figure 3:**
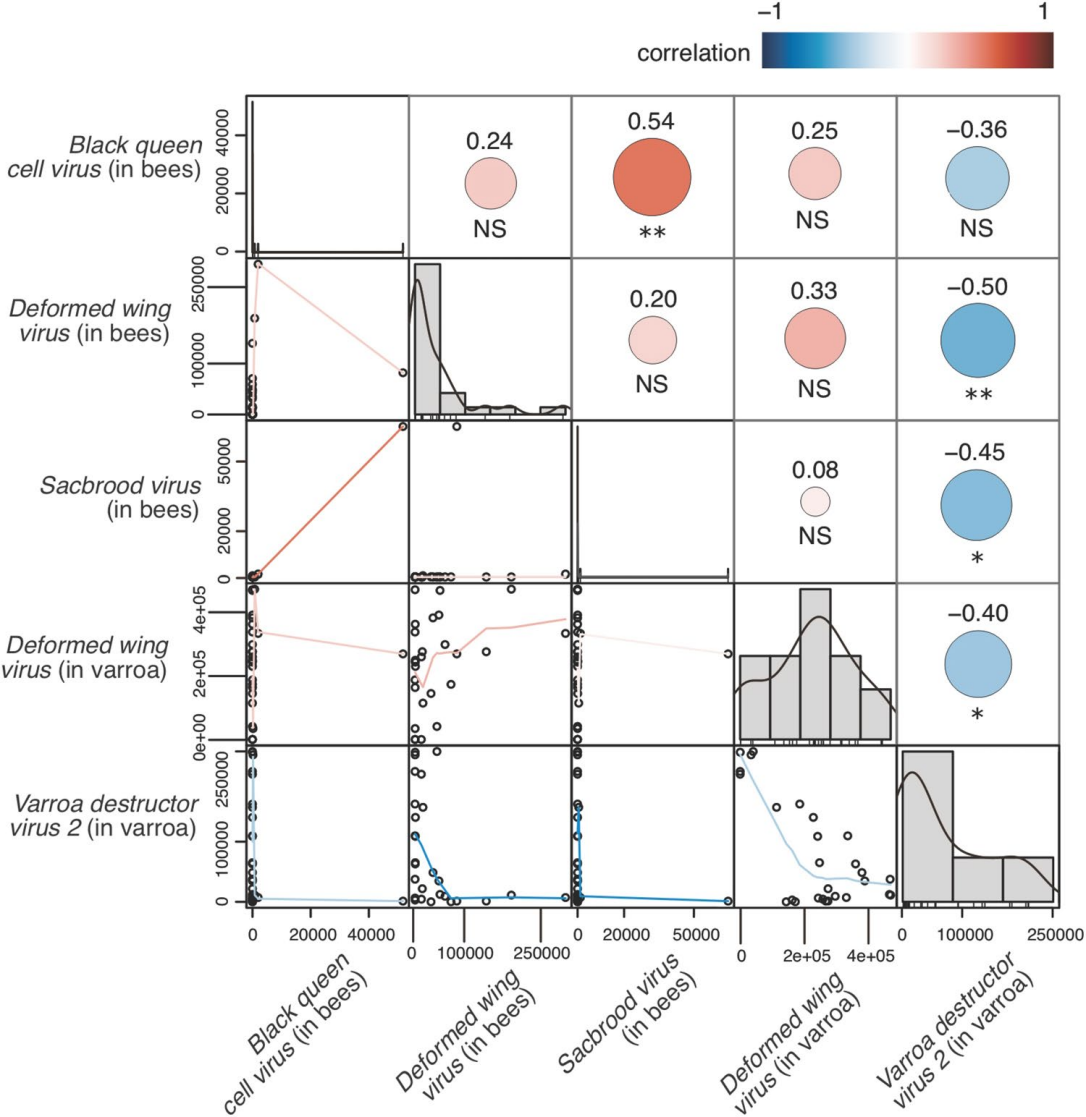
Pairwise correlations between virus loads in honey bees and *V. destructor*. The *V. destructor* and bee samples were paired, taken from the same hive. The size of the circles is representative of the Spearman correlation coefficient, which is shown above each circle with statistical significance indicated below. Only viruses that were identified in the PCA as important in explaining the variation in the communities were used in the analysis. * *p* < 0.05; ** *p* < 0.01. The counts are in transcripts per million.

Our pairwise comparisons focused on the viruses identified as key contributors in the PCAs, since we were interested only in associations between the highly abundant viruses. We therefore selected DWV-A, SBV and BQCV for further scrutiny in bees, plus DWV-A and VDV-2 in *V. destructor*. Specifically, in the analysis of individual hive replicates, where there were high levels of VDV-2 in mites, reduced DWV-A occurred in both the mites (Spearman’s correlation: *r*_*s*_ = − 0.404, *p* = 0.037; linear regression: *β* = − 1.145, *r*^*2*^ = 0.515, *p* < 0.001; Fig. 3, Supplementary Fig. S1) and the bees co-occurring within the same hive (*r*_*s*_ = − 0.498, *p* = 0.009; *β* = − 0.301, *r*^*2*^ = 0.155, *p* = 0.042; Fig. 3, Supplementary Fig. S1). Where there were high loads of DWV-A in mites, there were typically high loads in bees although the relationship was not statistically significant at a conventional 5% level (*r*_*s*_ = 0.334, *p* = 0.088; *β* = 0.163, *r*^*2*^ = 0.117, *p* = 0.081; Fig. 3, Supplementary Fig. S1). Strong correlations were observed for other pairs of viruses, including SBV and BQCV (Fig. 3), although given the extraordinarily low abundances of these viruses the associations are not likely to be biologically relevant.

### PCR and replication assays for selected RNA viruses

The presence of DWV-A, KBV and BQCV in the pooled bee and *V. destructor* samples was confirmed by RT-PCR, while VDV-2 and VDV-5 were only detected using RT-PCR in the pooled *V. destructor* sample (Table 2). The negative-sense, replicative strands of DWV-A and KBV were detected in both the pooled bee and *V. destructor* samples. We detected the negative-sense strand of VDV-2 and VDV-5 only in the pooled *V. destructor* samples. The negative-strand of BQCV was not detected in any of the samples using two different sets of tagged primers (Supplementary Table S4). IAPV and VDV-3 were not detected.

**Table 2:** RT-PCR and replication assay results for viruses in honey bees and *V. destructor*. Pooled *V. destructor* and honey bee samples were screened for eight viruses (DWV-A, DWV-B (VDV-1), BQCV, KBV, IAPV, VDV-2, VDV-3 and VDV-5). Positive test results are indicated with ( +) while (−) indicates that no band was observed. A positive result for the negative-strand detection assay is indicative that the virus is parasitising the host cells. Primer pairs used in this analysis are shown in Supplementary Table S4.

Virus or virus strain	RT-PCR detection		Negative-strand detection	
	Honey bee	V. destructor	Honey bee	V. destructor
DWV-A	+	+	+	+
DWV-B (VDV-1)	−	−	−	−
BQCV	+	+	−	−
KBV	+	+	+	+
IAPV	−	−	−	−
VDV-2	−	+	−	+
VDV-3	−	−	−	−
VDV-5	−	+	−	+

We used two sets of primers to examine for the presence of DWV-B/VDV-1. Firstly, specific primers (labelled DWV-B_L_F and DWV-B_L_R; Bradford et al.^57^) successfully amplified a 357 bp fragment in bee and *V. destructor* samples, however, a BLAST analysis of the sequenced PCR products revealed these to best match DWV-A. Testing these DWV-B/VDV-1 primers on the DWV-A reference genome (AY292384.1) in Geneious software showed that the DWV-B/VDV-1 forward primer could bind to DWV-A. In a second analysis, different DWV-B/VDV-1 primers (labelled DWV-B_F and DWV-B_R^57^), which targeted a shorter 116 bp region within the 357 bp region, were also tested against the DWV-A reference genome (AY292384.1) in Geneious, yet did not bind to any region. Likewise, PCR screening of *V. destructor* and bee samples using this second set of DWV-B/VDV-1 primers (DWV-B_F and DWV-B_R) failed to amplify any fragments in the samples.

### DNA virus confirmation assays

AmFV was found in the three pooled regional *V. destructor* samples and the three pooled regional bee samples (Supplementary Table S5). The two genes amplified in this analysis matched AmFV sequences in GenBank with > 97% identity. This DNA virus therefore appears to be present throughout New Zealand.

## Discussion

Our results indicate that it is likely that there has been only one introduction of *V. destructor* into New Zealand. An analysis of the mitochondrial gene cytochrome c oxidase I (MT-CO1) from six mites sampled from Auckland soon after the mites discovery in the year 2000^25^ indicated the presence of the Korean-like haplotype of *V. destructor*^56^. Unfortunately, these samples are not available for sequence comparison. One sequence from one mite was available on GenBank from a collection in 2016 in Kaikohe, in the upper North Island of New Zealand, that consisted of a 811 bp fragment of MT-CO1^55^. In the same study an additional mite was sampled from Gisborne (approximately 520 km from Kaikohe), which shared the exact same haplotype sequence. These samples also shared identical MT-CO1 sequences to our samples. While we cannot rule out the possibility of multiple introductions from a genetically similar source, only one invasion event for New Zealand seems likely given the higher genetic variation in *V. destructor* populations observed in other countries where multiple introductions have occurred (Fig. 1). Elsewhere, substantial genetic diversity in *V. destructor* populations has been found even within individual hives^58^. A high level of genetic diversity in countries such as Argentina has been hypothesised to allow spatial genetic differentiation of *V. destructor* populations due to different mite genotypes adapting to different climatic conditions^59^. A single introduction event to New Zealand will probably have limited the ability of *V. destructor* to adapt or evolve to environmental conditions, or potentially to evolve pesticide resistance. A single introduction event will also have limited the viral diversity and community introduced with this parasite.

DWV was likely to have been absent from New Zealand prior to the introduction of *V. destructor* in 2000^27^. All colonies that we sampled were positive for DWV-A in both *V. destructor* and bees, which was much higher than the 50% and 90% prevalence in a previous PCR-based study in New Zealand in 2014^27^. It is now typically the predominant virus present in both bees and mites. Mites were found to carry DWV-A loads that were on average 5.85-fold higher than in bees (as measured in transcripts per million reads). The RNA virome of several of our mites and bees samples had > 99% of the observed virus reads as DWV-A for both mites and bees, which is consistent with other studies^10,34,60^. Five of the 27 mite samples, however, had low numbers of DWV-A reads representing < 15% of the observed virome. In another study from Germany, many but not all mites in a population appear to carry DWV infections^61^. How and why some populations had such low DWV-A reads is unknown and worthy of more study. Our assays and other studies^62,63^ have found that DWV-A appears to replicate in both mites and bees, in contrast to more recent work where authors have concluded that this virus seems unable to replicate in mites^61,64^. We note the debate around using negative strand assays for DWV replication and that other approaches may be more definitive, as PCR approaches could give false-positive results after mites acquire negative strands from bees via their feeding^61^. We also observed no evidence of DWV-B (originally named VDV-1) from either the RNA-Seq or PCR analyses. DWV-B has become more prevalent and more virulent to honey bees in several countries^1,20,21^. The lack of this DWV-B is good news for beekeepers and may be due to the apparent single *V. destructor* introduction from a period prior to DWV-B becoming globally widespread, in addition to New Zealand’s complete ban on honey bee or honey bee product imports. Importing live bees continues to be a key contributing factor in emerging bee disease and colony loss elsewhere^65,66^.

The viral community in our bees from New Zealand was associated with that in *V. destructor*, which was previously observed in New Zealand bee and *V. destructor* communities^27^, but unlike in other studies such as Roberts et al.^67^ who analysed viruses in bees and *V. jacobsoni* in Papua New Guinea. The viruses observed in the bees examined by Roberts et al.^67^ formed a near completely different and distinct community compared to the mites that were parasitising the same hives. Bees in this region appear to tolerate mite infestations, which was attributed to a lack of DWV in mites and bees^67^. Similarly, honey bees on the remote island of Fernando de Noronha (Brazil) also tolerate *V. destructor* infestations, possibly because of extremely low DWV levels^68^. DWV is known to have an immunosuppressive effect that serves to enhance the reproduction and fitness of the parasitic mite^6^. Perhaps due to these immunosuppressive effects when DWV is prevalent, viral communities in bees and *V. destructor* become substantially more similar. The introduction of *V. destructor* and its associated DWV strains can alter the viral landscape of bees^17^ and even their predators^18^. These studies suggest that DWV plays a central role in not only influencing bee health but also the entire viral communities in *V. destructor*, bee hosts, and other insects.

Our analysis showed that after DWV-A, BQCV and SBV contributed most to the variance observed in the viral communities in honey bees. All but two of our 27 honey bee samples were positive for BQCV, and 22 were positive for SBV. Both of these viruses were found in the *V. destructor* samples, but at substantially lower loads. These observed infection rates were consistent with previous work in New Zealand^27^. There is no evidence that *V. destructor* can vector either SBV or BQCV^1^, but there is evidence that SBV can modify mite behaviour after it becomes infected as a result of mite feeding^37^. Of note for our samples was the near absence of CBPV, which was present at extremely low levels in only three of the 27 honey bee samples and absent from mites. CBPV was present in 20–40% of samples from one previous NZ study^27^, but a more recent analysis showed apiaries have infection rates < 20%^26^. This virus has been identified as a major emerging threat to honey bees elsewhere^65^.

VDV-2 and VDV-5 were found in both bees and *V. destructor* in our RNA-Seq analysis. The read counts were on average 7,441-fold and 21-fold higher in *V. destructor* than in bees, respectively. Other researchers have observed similar results to ours, with both present but without evidence of replication in bees^11,69^. Their presence in bees seems to be related to feeding by the mite^3^ wherein they acquire a small number of viral particles. Such a small number of particles probably explains why we observed both VDV-2 and VDV-5 in bees using the RNA-Seq analysis, but not in PCR approaches. Our assembled contigs for VDV-2 and VDV-5 showed 85–92% nucleotide identity compared to published genome sequences. This suggests that the sequences are clearly variants of VDV-2 and VDV-5. There was, however, considerable strain diversity, particularly in the VDV-2 strains. This level of diversity was surprising given the probable single introduction of *V. destructor* to New Zealand and is currently under further investigation.

Seventeen RNA viruses were observed in honey bees and 10 in *V. destructor.* These viruses represent a subset of the 87 and 59 viruses that have previously been described to infect honey bees^9^ and *V. destructor* (Supplementary Table S1), respectively. Several viruses, including IAPV, were not found in our analyses and have been concluded as absent from New Zealand in previous studies that have used PCR-based assays^26–28^. Many of the viruses that we observed in honey bees were prevalent in only a few samples or were in low abundance. KBV was present in 66% of bee samples and 33% of mites, typically in extremely low relative abundance. These KBV prevalence rates are similar to other work on New Zealand honey bees^26,27^. Mondet et al.^27^ observed that KBV peaked in abundance 2 years after *V. destructor* invasion but appeared to then disappear from colonies entirely and be replaced by DWV. Our results with extremely low titre of KBV are consistent with this pattern. In contrast, our work on KBV in invasive wasps from New Zealand demonstrated infection in every single individual from every nest we examined, incurring fitness costs for the wasps^70^. We also found three *Lake Sinai virus* (LSV) strains, but only low relative abundance in honey bees. Other researchers have described LSV from *V. destructor*^71^. In addition to these RNA viruses, the large double stranded DNA virus of honey bees, *Apis mellifera filamentous virus* (AmFV), was observed in mites and bees. This virus may be ubiquitous in honey bees^72^. It has been described as weakly pathogenic to honey bees, but it can alter bee behaviour and physiology when in high abundance^73^. AmFV has previously been observed in other bee species^73^ and *V. destructor* mites^36^.

Our RNA-Seq approach tentatively indicated a diversity of other viruses or viral strains. We excluded these species from the analysis presented here due to low confidence in their similarity to known viruses. More work is needed to define and describe many of these viral species that were typically rare and in extremely low relative abundance. We strongly suspect there were more than just 10 virus species present in *V. destructor* and in the honey bees, especially as at least 59 putative viruses have previously been described from this invasive mite (Supplementary Table S1). Our RNA-Seq data can be explored further with the potential to describe new viral species, strains, and host associations. It is also possible that additional sampling within New Zealand would identify additional viruses, or even that additional sampling might identify further haplotypes of *V. destructor.*

Our results also suggest a relationship between interspecific viral interactions within mites, which influences those interactions in bees. Specifically, where we observed high levels of VDV-2 in mites, reduced DWV-A occurred in both the mites and the bees co-occurring within the same hive. Where there were high loads of DWV-A in mites, there were typically high loads in bees. Perhaps as both VDV-2 and DWV-A belong to the Iflaviridae viral family, they may compete for similar resources within host cells. There is evidence of competition between viruses in honey bees from other studies^74^, with this virus-virus competition potentially mediating colony collapse^75^. Other work has found that the DWV load in mites is dynamic and can rapidly increase or decrease depending on the DWV load of the honey bees upon which they are feeding^64^. Overt infections of DWV-A might only occur after the viral titre has exceeded a certain threshold^62^, with VDV-2 restraining DWV-A infections. We do note, however, that such a relationship between viruses such as DWV and VDV-2 was not observed in *V. destructor* examined in Herrero et al.^69^. Experimental approaches are needed to better understand how the viral community in *V. destructor* and honey bees interact.

We found no evidence of different viral communities occurring in mites or bees in the different regions of New Zealand. Mondet et al.^27^ suggested that a ‘dynamic and turbulent pathological landscape’ forms that settles into a more stable and predictable pattern 2- 3 years after *V. destructor* invasion. Our results are consistent with their hypothesis. We did, however, still observe high levels of variation within regions. Understanding the mechanisms for this variation may help beekeepers cope with what appears to be a worsening *V. destructor* problem.

## Supplementary Information

Supplementary Information.

## Data Availability

Clean reads from which we assembled and quantified viral transcripts can be accessed from the NCBI SRA repository at http://www.ncbi.nlm.nih.gov/bioproject/820512 under BioProject PRJNA820512.
