# Supplementary material for: Viral communities in the parasite Varroa destructor and in colonies of their honey bee host (Apis mellifera) in New Zealand
Source: Sci Rep. 2022 May 25;12:8809. doi: 10.1038/s41598-022-12888-w (PMC9133037; doi:10.1038/s41598-022-12888-w)
Supplement: Supplementary file 1 — Supplementary Information. [file 41598_2022_12888_MOESM1_ESM.pdf]

Supplementary Information for

Viral communities in the parasite *Varroa destructor* and in colonies of their honey bee host (*Apis mellifera*) in New Zealand

Philip J. Lester[1,*,+] , Antoine Felden[1,+], James W. Baty[1], Mariana Bulgarella[1], John Haywood[2], Ashley N. Mortensen[3], Emily J. Remnant[4] & Zoe E. Smeele[1]

[1]Centre for Biodiversity and Restoration ecology, School of Biological Sciences, Victoria University of Wellington, PO Box 600, Wellington, 6012, New Zealand. [2]School of Mathematics and Statistics, Victoria University of Wellington, PO Box 600, Wellington, 6012, New Zealand. [3]The New Zealand Institute for Plant and Food Research Limited, Private Bag 3230, Waikato Mail Centre, Hamilton 3240, New Zealand. [4]Behaviour, Ecology and Evolution Laboratory, School of Life and Environmental Sciences, Science Road, University of Sydney, Sydney, NSW 2006, Australia. *Correspondence and requests for materials should be addressed to P.J.L. (email: Phil.Lester@vuw.ac.nz) or A.F. (email: Antoine.Felden@vuw.ac.nz). +these authors contributed equally to this work

This PDF file includes:

Figure S1
Tables S1 to S9
SI References

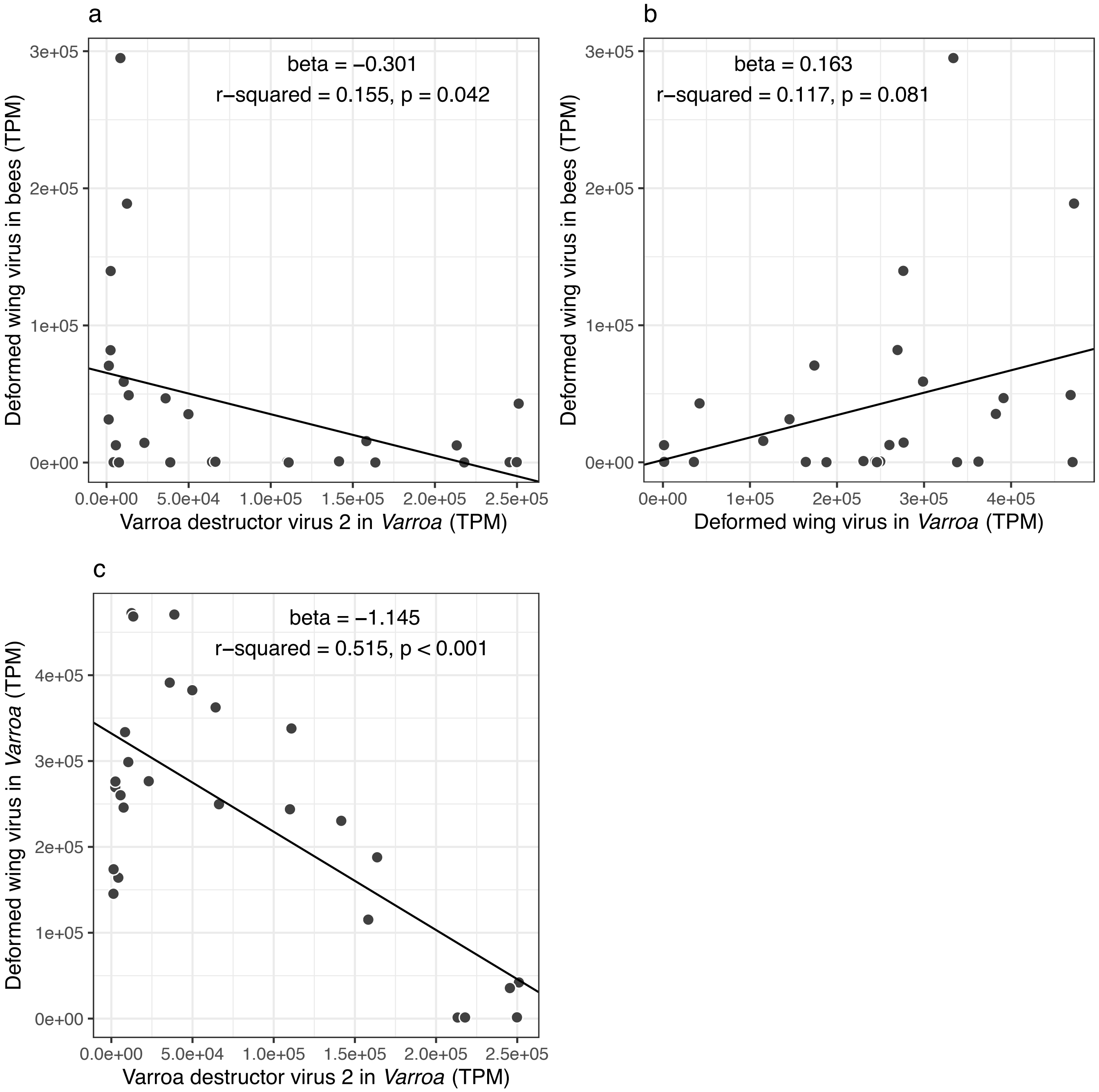

Supplementary Figure S1. Linear regressions between (a) *Varroa destructor virus 2* (VDV-2) in *V. destructor* and the *Deformed wing virus* (DWV-A) in bees, (b) DWV-A in *V. destructor* and DWV in bees and (c) VDV-2 and DWV in *V. destructor*.

Supplementary Table S1. A list of putative virus species reported from *V. destructor* using RNA-Seq, genomic or PCR based approaches. A total of 59 putative species have been reported, but the taxonomic certainty varies considerably between species and studies. The similarity values are for nucleotide identities in %, unless when AA is specified, indicating amino acid sequence similarity.

Genome	Order	Family	Virus	Acronym	Similarity†	Location	Reference*
**RNA viruses**							
ssRNA(-)	Articulavirales	Orthomyxoviridae	*Aransas Bay virus*	ABV	78%	Israel	Levin et al. (2016)
ssRNA(-)	Articulavirales	Orthomyxoviridae	*Dhori virus*	DHOV	56%, 53%	Algeria	Haddad et al. (2018), Levin et al. (2016)
ssRNA(-)	Articulavirales	Orthomyxoviridae	*Jos virus*	JOSV	59%, 72%	Israel, Jordan	Haddad et al. (2018), Levin et al. (2016)
ssRNA(-)	Articulavirales	Orthomyxoviridae	*Thogoto virus*	THOV	44%	Jordan	Haddad et al. (2018)
ssRNA(-)	Articulavirales	Orthomyxoviridae	*Tjuloc virus*	TLV	39%	Jordan	Haddad et al. (2018)
ssRNA(-)	Articulavirales	Orthomyxoviridae	*Varroa orthomyxovirus-1*	VOV-1	N.S.	Algeria, Israel, Jordan	Levin et al. (2019)
ssRNA(-)	Articulavirales	Orthomyxoviridae	*Wellfleet Bay virus*	WFBV	39%	Algeria	Haddad et al. (2018)
ssRNA(-)	Mononegavirales	Rhabdoviridae	*Apis rhabdovirus 1/Bee rhabdovirus*	ARV-1/BRV-1	?, ?	Netherlands, South Africa, Algeria, Israel, Jordan	Remnant et al. (2017), Levin et al. (2019), this study
ssRNA(-)	Mononegavirales	Rhabdoviridae	*Apis rhabdovirus 2*	ARV-2	?, ?	Netherlands, South Africa, Algeria, Israel, Jordan, New Zealand	Remnant et al. (2017), Levin et al. (2019), this study
ssRNA(-)	Mononegavirales	Rhabdoviridae	*Farmington virus*	FARV	45%	Algeria, Jordan	Haddad et al. (2018)
ssRNA(-)	?	?	*Hubei picorna-like virus 22*		71-73%[AA]	New Zealand	This study
ssRNA(-)	?	?	*Hubei picorna-like virus 29*			Thailand	Levin et al. (2019)
ssRNA(-)	?	?	*Jingshan Fly Virus 2*		52%	Algeria	Haddad et al. (2018)
ssRNA(+)	Nodamuvirales	Nodaviridae	*Mosinovirus*	MoNV	41%	Jordan	Haddad et al. (2018)
ssRNA(+)	Picornavirales	Dicistroviridae	*Acute bee paralysis virus*	ABPV / IAPV	?, ?	Algeria, Israel, Thailand	Chantawannakul et al. (2006), Levin et al. (2019),
ssRNA(+)	Picornavirales	Dicistroviridae	*Black queen cell virus*	BQCV	?, 99%, 97%, ?, 99%	Algeria, Israel, Jordan, New Zealand, Thailand	Chantawannakul et al. (2006), Haddad et al. (2018), Levin et al. (2016), Levin et al. (2019), Mondet et al. (2014), this study
ssRNA(+)	Picornavirales	Dicistroviridae	*Israeli acute paralysis virus*	IAPV	83%	Israel	Levin et al. (2016)
ssRNA(+)	Picornavirales	Dicistroviridae	*Kashmir Bee Virus*	KBV	99%	New Zealand	Mondet et al. (2014), this study

ssRNA(+)	Picornavirales	Iflaviridae	*Antheraea pernyi iflavirus*	AVD	54%	Israel	Levin et al. (2016)
ssRNA(+)	Picornavirales	Iflaviridae	*Deformed wing virus A*	DWV	?, 98%, 97%, ?, 98%	Algeria, Israel, Jordan, New Zealand, Thailand	Chantawannakul et al. (2006), Haddad et al. (2018), Levin et al. (2016), Levin et al. (2019), Mondet et al. (2014), this study
ssRNA(+)	Picornavirales	Iflaviridae	*Deformed wing virus B / Varroa destructor virus 1*	DWV-B / VDV-1	98%, 97%	Algeria, Israel	Haddad et al. (2018), Levin et al. (2016)
ssRNA(+)	Picornavirales	Iflaviridae	*Formica exsecta virus*	FeV	N.S.	Israel	Levin et al. (2016)
ssRNA(+)	Picornavirales	Iflaviridae	*Formica exsecta virus 2*	FeV2	48%, 56%	Algeria, Jordan	Haddad et al. (2018), Levin et al. (2016)
ssRNA(+)	Picornavirales	Iflaviridae	*Halyomorpha halys virus*	HhV	45%, 56%	Israel, Jordan	Haddad et al. (2018), Levin et al. (2016)
ssRNA(+)	Picornavirales	Iflaviridae	*Heliconius erato iflavirus*	HeIV	51%, 58%	Algeria, Israel	Haddad et al. (2018), Levin et al. (2016)
ssRNA(+)	Picornavirales	Iflaviridae	*Laodelphax striatellus picorna-like virus 2*	LsPV2	47%	Jordan	Haddad et al. (2018)
ssRNA(+)	Picornavirales	Iflaviridae	*Sacbrood virus*	SBV	?, 96%, 99%, 99%	Algeria, Israel, Jordan, New Zealand, Thailand	Chantawannakul et al. (2006), Haddad et al. (2018), Levin et al. (2016), Mondet et al. (2014), this study
ssRNA(+)	Picornavirales	Iflaviridae	*Slow bee paralysis virus*	SBPV	50%, 99%	Algeria, Israel, Jordan	Haddad et al. (2018), Levin et al. (2016)
ssRNA(+)	Picornavirales	Iflaviridae	*Spodoptera exigua iflavirus 1*	SeIV-1	50% 64%	Algeria, Israel, Jordan	Haddad et al. (2018), Levin et al. (2016)
ssRNA(+)	Picornavirales	Iflaviridae	*Thaumetopoea pityocampa iflavirus 1*	TpIV-1	42%	Algeria	Haddad et al. (2018)
ssRNA(+)	Picornavirales	Iflaviridae	*Varroa destructor virus 2*	VDV-2	83%, 96%, 83%, N.S., ?	Algeria, China, Israel, Jordan, Thailand, UK	Chen et al. (2021), Haddad et al. (2018), Herrero et al. (2019), Levin et al. (2016), Levin et al. (2019), this study
ssRNA(+)	Picornavirales	Picornaviridae	*Bat feces associated picorna-like virus*		50%	Israel	Levin et al. (2016)
ssRNA(+)	Picornavirales	Picornaviridae	*Picorna-like virus Eptesicus fuscus*		41%	Jordan	Haddad et al. (2018)
ssRNA(+)	Picornavirales	?	*Bundaberg bee virus 1*	BBV-1	77%[AA]	New Zealand	This study
ssRNA(+)	Tymovirales	Tymoviridae	*Bee/Varroa destructor Macula-like virus*	BeeMLV/ VdMLV	90%	Algeria, Israel, Jordan	Levin et al. (2019)

ssRNA(+)	Tymovirales	Tymoviridae	*Erysimum latent virus*	ELV	62%	Algeria	Haddad et al. (2018)
ssRNA(+)	Tymovirales	Tymoviridae	*Grapevine Red Globe virus*	GRGV	74%	Jordan	Haddad et al. (2018)
ssRNA(+)	Tymovirales	Tymoviridae	*Physalis mottle virus*	PhMV	70%	Algeria, Jordan	Haddad et al. (2018)
ssRNA(+)	Tymovirales	Tymoviridae	*Plantago mottle virus*	PlMV	53%	Algeria	Haddad et al. (2018)
ssRNA(+)	Tymovirales	Tymoviridae	*Scrophularia mottle virus*	SCRMV0	69%	Algeria, Jordan	Haddad et al. (2018)
ssRNA(+)	Tymovirales	Tymoviridae	*Tomato blistering mosaic virus*	ToBMV	57%	Algeria	Haddad et al. (2018)
ssRNA(+)	Tymovirales	Tymoviridae	*Turnip yellow mosaic virus*	TYMV	65%	Jordan	Haddad et al. (2018)
ssRNA(+)	Tymovirales	Tymoviridae	*Varroa destructor Macula-like virus*	VdMLV	78%	Algeria, Jordan	Haddad et al. (2018)
ssRNA(+)	Tymovirales	Tymoviridae	*Watercress white vein virus*	WWVV	48%	Algeria	Haddad et al. (2018)
ssRNA(+)	?	?	*Chronic bee paralysis virus*	CBPV	99%	New Zealand	Mondet et al. (2014)
ssRNA(+)	?	?	*Varroa destructor virus 3*	VDV-3	95%, 91%, N.S., 98%,	Algeria, Israel, Jordan, USA	Haddad et al. (2018), Herrero et al. (2019), Levin et al. (2016), Levin et al. (2019)
ssRNA(+)	?	?	*Varroa destructor virus 5*	VDV-5		UK	Herrero et al. (2019), this study
**DNA viruses**							
dsDNA	Lefavirales	Baculoviridae	*Epiphyas postvittana nucleopolyhedrovirus*	EppoNPV	68%	Israel	Levin et al. (2016)
dsDNA	?	?	*Apis mellifera filamentous virus*	AmFV		USA	Gauthier et al. (2015)
ssDNA	Cirlivirales	Circoviridae	*Anguilla anguilla circovirus*		77%	Israel	Levin et al. (2016)
ssDNA	Cirlivirales	Circoviridae	*Bat circovirus ZS/China*		77%	Algeria, Jordan	Haddad et al. (2018)
ssDNA	Cirlivirales	Circoviridae	*Cyclovirus*		60%, ?	Algeria, Israel	Levin et al. (2016), Levin et al. (2019)
ssDNA	Cirlivirales	Circoviridae	*Cyclovirus NG14*		65%	Jordan	Haddad et al. (2018)
ssDNA	Cirlivirales	Circoviridae	*Cyclovirus NGchicken8*		60%	Jordan	Haddad et al. (2018)
ssDNA	Cirlivirales	Circoviridae	*Cyclovirus TN18*		50%	Algeria, Jordan	Haddad et al. (2018)
ssDNA	Cirlivirales	Circoviridae	*Cyclovirus TN25*		61%	Algeria, Jordan	Haddad et al. (2018)
ssDNA	Cirlivirales	Circoviridae	*Dragonfly cyclovirus 1*	DfCyV-1	59%	Jordan	Haddad et al. (2018)
ssDNA	Cirlivirales	Circoviridae	*Dragonfly cyclovirus 4*	DfCyV-4	46%	Algeria, Jordan	Haddad et al. (2018)
ssDNA	Cirlivirales	Circoviridae	*Dragonfly cyclovirus 6*	DfCyV-6	64%	Algeria, Jordan	Haddad et al. (2018)
**Unclassified virus**							
?	?	?	*Beihai horseshoe crab virus-1*		?	Algeria	Levin et al. (2019)

?	?	?	*Varroa destructor virus 4*	VDV-4	N.S.	Thailand	Levin et al. (2019)

† The Similarity column represents % nucleotide sequence identities. In some publications similarity values are given for multiple genes or sequences, under which circumstances we choose the highest value from all sequences given. N.S. = New Species, or a virus that was named and described within the publication; ? = similarity values were not given in the manuscript.

* Full references are:

Chantawannakul, P., et al. (2006). "A scientific note on the detection of honeybee viruses using real-time PCR (TaqMan) in *Varroa* mites collected from a Thai honeybee (*Apis mellifera*) apiary." Journal of Invertebrate Pathology 91(1): 69-73.

Chen, G., et al. (2021). "A new strain of virus discovered in China specific to the parasitic mite *Varroa destructor* poses a potential threat to honey bees." Viruses 13(4): 679.

Haddad, N., et al. (2018). "Next-generation sequence data demonstrate several pathogenic bee viruses in Middle East and African honey bee subspecies (*Apis mellifera syriaca*, *Apis mellifera intermissa*) as well as their cohabiting pathogenic mites (*Varroa destructor*)." Virus Genes 54(5): 694-705.

Herrero, S., et al. (2019). "Identification of new viral variants specific to the honey bee mite *Varroa destructor*." Experimental and Applied Acarology 79(2): 157-168.

Levin, S., et al. (2016). "Two novel viruses associated with the *Apis mellifera* pathogenic mite *Varroa destructor*." Scientific Reports 6: 37710.

Levin, S., et al. (2019). "New viruses from the ectoparasite mite *Varroa destructor* infesting *Apis mellifera* and *Apis cerana*." Viruses 11(2): 94.

Mondet, F., et al. (2014). "On the front line: quantitative virus dynamics in honeybee (A*pis mellifera* L.) colonies along a new expansion front of the parasite *Varroa destructor*." PLoS Pathogens 10(8): e1004323.

Remnant, E. J., et al. (2017). "A diverse range of novel RNA viruses in geographically distinct honey bee populations." Journal of Virology 91(16): e00158-00117.

Supplementary Table S2. Study site locations. The "file name" columns refer to the clean reads deposited on the NCBI SRA server.

**Area**	Number (on Fig. 2)	Sample identification (ID) for supplementary tables, below	Approximate location	Latitude	Longitude	File name for bee reads	File name for varroa reads
South	i	si	Dunedin	-45.86	170.50	B1	V1
South	ii	sii	Ashburton	-43.64	171.42	B2	V2
South	iii	siii	Ashburton	-43.64	171.42	B3	V3
South	iv	siv	Ashburton	-43.67	171.41	B4	V4
South	v	sv	Ashburton	-43.67	171.41	B5	V5
South	vi	svi	Ashburton	-43.86	171.96	B6	V6
South	vii	svii	Ashburton	-43.93	171.78	B7	V7
South	viii	sviii	Ashburton	-43.87	171.83	B8	V8
South	ix	six	Ashburton	-43.87	171.83	B9	V9
Central	i	ci	Porirua, Wellington	-41.14	174.86	B10	V10
Central	ii	cii	Otaki	-40.76	175.16	B11	V11
Central	iii	ciii	Kelburn, Wellington	-41.29	174.77	B12	V12
Central	iv	civ	Kelburn, Wellington	-41.29	174.77	B13	V13
Central	v	cv	Feilding	-40.14	175.65	B15	V15
Central	vi	cvi	Wadestown, Wellington	-41.26	174.77	B16	V16
Central	vii	cvii	Wadestown, Wellington	-41.26	174.77	B17	V17
Central	viii	cviii	Vogeltown, Wellington	-41.31	174.77	B18	V18
North	i	ni	Matangi, Hamilton	-37.80	175.33	B20	V20
North	ii	nii	Tamahere, Hamilton	-37.83	175.35	B21	V21
North	iii	niii	Horotiu, Hamilton	-37.70	175.20	B22	V22
North	iv	niv	Horotiu, Hamilton	-37.70	175.20	B23	V23
North	v	nv	Horotiu, Hamilton	-37.70	175.20	B24	V24
North	vi	nvi	Enderly, Hamilton	-37.77	175.31	B25	V25
North	vii	nvii	Enderly, Hamilton	-37.77	175.31	B26	V26
North	viii	nviii	Matangi, Hamilton	-37.81	175.40	B27	V27
North	ix	nix	Matangi, Hamilton	-37.81	175.40	B28	V28
North	x	nx	Tamahere, Hamilton	-37.83	175.35	B29	V29

Supplementary Table S3. Sample information and GenBank accession numbers for individual *V. destructor* mite sequenced and used in this study to build a phylogenetic tree. All localities occur in New Zealand.

Sample ID	Locality	Sample codes	GenBank Accession MT-CO1 (727 bp)	GenBank Accession MT-CO3 (339 bp)	GenBank Accession MT-ATP6 (166 bp)
nv	Horotiu, Hamilton		OK560013	OK667045	OK667056
sv	Ashburton		OK560014	OK667046	OK667057
ci	Porirua, Wellington		OK560015	OK667047	OK667058
cv	Feilding		OK560016	OK667048	OK667059
nviii	Matangi, Hamilton		OK626252	N/A	N/A
nii	Tamahere, Hamilton		OK626253	OK667049	OK667060
niv	Horotiu, Hamilton		OK626254	OK667050	OK667061
nvi	Enderley, Hamilton		OK626255	OK667051	OK667062
cii	Otaki		OK626256	OK667052	OK667063
ciii	Kelburn, Wellington		OK626257	OK667053	OK667064
si	Ashburton		OK626258	OK667054	OK667065
sii	Ashburton		OK626259	OK667055	OK667066
siii	Ashburton		OK626260	N/A	N/A

Supplementary Table S4. PCR primers used in this study.

Name	Sequence (5'-3')	Product size (bp)	Reference
DWV-F15	TCCATCAGGTTCTCCAATAACGGA	451	Yue & Genersch (2005)[1]
DWV-B23	CCACCCAAATGCTAACTCTAAGCG		
DWV Tag-F15 (replication)	agcctgcgcaccgtggTCCATCAGGTTCTCCAATAACGGA		
Tag	agcctgcgcaccgtgg		
DWV-A_L_F	GTACTCTCCTATATCAGTTTCG	409	Bradford et al. (2017)[2]
DWV-A_L_R	TTTTACGCTCACCGGCGCT		
DWV-B_L_F	CGAATTACGGTGCAACTAAC	357	Bradford et al. (2017)[2]
DWV-B_L_R	GTCACATGGCATTCTACTCT		
DWV-A_F	GCGTGTTGCAACTCGCTTC	211	Bradford et al. (2017)[2]
DWV-A_R	TGCCTGCACCGGATTCGATAAT		
DWV-B_F	GCAAGTTGGAGATAATTGTA	116	Bradford et al. (2017)[2]
DWV-B_R	CGATACTTACATTCTTCAAGAT		
Pan-DWV_F	ACGCAACCCCAGGAAT	375	Bradford et al. (2017)[2]
Pan-DWV_R	GTAGCTAATTTTACCCAATCTTTAAA		
VDV-1_FRT	GCGAAGTAGAATTTACTTCTTCA	1322	Ongus et al. (2004)[3]
VDV-1_R	AGCACGAGCATGTTCAGC		
VDV-1_FRTPCR	CGAAACGAAGAGAGCATGTAT	1129	Ongus et al. (2004)[3]
VDV-1_RRTPCR	CGACTCTTCCCCAGCTAAG		
VDV-2_UK_F	ATCCAGATTTGGAGGAGGTG	150	Herrero et al. (2019)[4]
VDV-2_UK_R	TCATCGAGACAATCCTCGTC		
neg_VDV-2_F (replication)	ggatgcaggctacgtgaagatacg - CGTCTCCATGCAGAATTAGCTGG	1987	
TagF	ggatgcaggctacgtgaagatacg		
VDV-3_USA_F	TCGAACGACCTGAAGAGAAG	121	Herrero et al. (2019)[4]
VDV-3_USA_R	GATGGGCATCTGATCATTCC		
VDV-5-F	AGACGTGCTCTTGAGATGGAG	74	Herrero et al. (2019)[4]
VDV-5-R	TCGCGCTTAGCTTCTTTCTC		
neg_VDVs_F (replication VDV3 and VDV-5)	ggatgcaggctacgtgaagatacg - AGATCAATCTGTAATAGATTGAC	506	Herrero et al. (2019)[4]
TagF	ggatgcaggctacgtgaagatacg	233	
BQCV_3UTR-F	TGGTCAGCTCCCACTACCTTAAAC	700	Singh et al. (2010)[5], Benjeddou et al. (2001)[6]
BQCV_3UTR-R	GCAACAAGAAGAAACGTAAACCAC		
BQCV_3UTR-tag-F (replication)	agcctgcgcaccgtggTGGTCAGCTCCCACTACCTTAAAC		
Tag	agcctgcgcaccgtgg		
Tag-BQCV-sense	agcctgcgcaccgtggTCAGGTCGGAATAATCTCGA	420	Peng et al. (2011)[7]
Tag	agcctgcgcaccgtgg		
BQCV-antisense	GCAACAAGAAGAAACGTAAACCAC		
KBV_capsid_F	TGTTTGTGGCAATCCAGCTA	625	Singh et al. (2010)[5], Benjeddou et al. (2001)[6]
KBV_capsid_R	TACGTCTTCTGCCCATTTCC		
KBV_capsid_tag-F (replication)	agcctgcgcaccgtggTGTTTGTGGCAATCCAGCTA		
Tag	agcctgcgcaccgtgg		
IAPV_L17M_F	CGAACTTGGTGACTTGAAGG	113	Palacios et al. (2008)[8]
IAPV_R130M_R	ACGTCAGTCGTCTTCCAGGT		

Supplementary Table S5. Sample information and GenBank accession numbers for the pooled mite and pooled bee samples used to screen for *Apis mellifera filamentous virus* (AmFV) via PCR. Note that for the bees, AmFV was present in all three New Zealand regions screened but we only Sanger sequenced samples from one region to confirm identity.

**Sample species and geographic origin**	**Genbank accession numbers for *Apis mellifera* filamentous virus thymidylate synthase gene**	**Genbank accession numbers for *Apis mellifera* filamentous virus ribonucleotide reductase small subunit gene**
1 – *Varroa destructor* from 9 beehives in the upper North Island of New Zealand	OL348207	Not submitted
2 – *Varroa destructor* from 8 beehives in the lower North Island of New Zealand	OL348208	OL348205
3 – *Varroa destructor* from 9 beehives in the South Island of New Zealand	OL348209	OL348206
Apis mellifera from 9 beehives in the lower North Island of New Zealand	OL356345	OL348204

Supplementary Table S6. A list of virus species detected in honey bee samples using RNA-Seq, given as the percentage of total viral loads. We report a total of 17 viruses with amino acid sequence identity >70% using DIAMOND BLAST, verified using BLASTn.

Sample ID	*Aphid lethal paralysis virus*	*Apis rhabdovirus 1*	*Apis rhabdovirus 2*	*Black queen cell virus*	*Bundaberg bee virus 2*	*Chronic bee paralysis virus*	*Deformed wing virus*	*Drosophila subobscura Nora virus*	*Hobart bee virus 1*	*Hubei picorna-like virus 15*	*Kashmir bee virus*	*Lake Sinai virus 1*	*Lake Sinai virus 3*	*Sacbrood virus*	*Varroa destructor virus 2*	*Varroa destructor virus 5*	*Vespa velutina Moku virus*
si	-	<0.01	-	<0.01	-	-	99.95	0.02	-	-	<0.01	-	-	-	0.02	-	<0.01
sii	-	34.3	0.02	0.31	-	-	64.34	-	-	-	0.01	-	-	0.03	1	-	-
siii	-	2.53	0.01	<0.01	-	-	97.43	-	-	-	-	-	-	-	0.02	-	-
siv	-	0.1	-	0.54	-	-	95.5	-	-	-	-	-	-	-	3.86	-	-
sv	-	56.05	0.02	-	-	-	30.2	-	-	-	-	1.51	10.53	0.02	1.29	0.26	0.12
svi	-	<0.01	-	<0.01	-	-	99.97	-	-	-	-	-	-	<0.01	0.03	-	-
svii	-	<0.01	-	<0.01	-	-	99.95	-	-	-	-	-	-	<0.01	0.05	-	-
sviii	-	0.06	-	39.94	-	-	55.44	-	0.66	0.24	0.03	-	-	0.12	3.51	-	-
six	-	0.02	-	3.99	3.12	0.01	79.12	-	3.65	-	0.08	0.01	-	6.15	3.84	-	-
ci	-	<0.01	-	25.9	-	-	41.03	-	-	<0.01	0.67	<0.01	-	32.4	<0.01	-	-
cii	0.01	0.04	<0.01	0.65	-	-	98.83	-	-	<0.01	<0.01	0.06	-	0.4	<0.01	<0.01	-
ciii	-	<0.01	-	0.04	-	0.32	99.58	-	-	-	<0.01	-	-	0.05	0.01	-	-
civ	-	0.31	<0.01	0.02	-	-	99.6	-	-	-	<0.01	<0.01	-	0.03	0.04	-	-
cv	<0.01	<0.01	-	0.4	-	-	99.56	-	-	<0.01	<0.01	0.03	-	<0.01	<0.01	-	<0.01
cvi	-	<0.01	-	0.02	-	-	99.91	-	-	-	<0.01	<0.01	-	0.07	0.01	-	-
cvii	-	0.19	<0.01	0.02	-	-	99.75	-	-	-	<0.01	-	-	0.03	0.01	-	-

cviii	-	0.03	-	0.11	-	-	94.42	-	-	-	0.02	-	-	0.08	0.75	-	4.59
ni	0.27	73.54	-	0.97	-	-	22.83	-	0.05	0.14	0.01	-	-	2.17	0.01	-	-
nii	-	0.06	-	1.62	-	-	93.59	-	-	0.1	-	-	-	-	1.75	2.88	-
niii	-	1.9	0.03	<0.01	-	-	95.05	-	-	<0.01	-	-	-	2.96	0.06	-	-
niv	-	88.58	0.02	0.86	-	-	7.12	-	-	2.63	0.01	-	-	0.1	0.69	-	-
nv	-	0.07	-	-	-	-	97.44	-	-	-	<0.01	0.05	-	0.08	2.35	-	-
nvi	0.24	-	-	0.07	-	0.01	99.61	-	-	-	-	-	-	-	-	-	0.08
nvii	-	<0.01	-	<0.01	-	-	99.97	-	-	-	-	-	-	<0.01	0.02	0.01	-
nviii	-	-	-	0.01	-	-	99.28	-	-	0.01	0.64	-	-	<0.01	0.06	-	-
nix	-	0.41	0.01	0.01	0.02	-	99.32	-	-	<0.01	<0.01	-	-	0.15	0.07	-	-
nx	0.37	0.02	-	0.51	-	-	47.17	-	-	-	0.02	-	-	49.69	2.23	-	-

Supplementary Table S7. A list of virus species detected in *V. destructor* samples using RNA-Seq, given as percentage of total viral loads. We report a total of 10 viruses with amino acid sequence identity >70% using DIAMOND BLAST, verified using BLASTn.

Sample ID	*Apis rhabdovirus 1*	*Apis rhabdovirus 2*	*Black queen cell virus*	*Bundaberg bee virus 2*	*Deformed wing virus*	*Hubei picorna-like virus 22*	*Kashmir bee virus*	*Sacbrood virus*	*Varroa destructor virus 2*	*Varroa destructor virus 5*
si	<0.01	<0.01	-	-	96.61	-	<0.01	-	3.38	0.01
sii	0.01	0.01	<0.01	-	68.85	-	-	<0.01	31.07	0.07
siii	0.02	0.02	-	-	0.59	-	-	<0.01	99.12	0.26
siv	0.03	0.02	<0.01	-	0.6	-	<0.01	-	99.28	0.08
sv	<0.01	<0.01	-	-	84.94	-	<0.01	<0.01	15.05	<0.01
svi	<0.01	<0.01	<0.01	-	92.28	-	-	-	7.69	0.03
svii	0.02	0.02	0.02	-	14.31	-	0.06	0.09	85.38	0.09
sviii	0.02	0.03	<0.01	-	12.63	-	-	<0.01	87.18	0.15
six	<0.01	<0.01	-	-	97.45	-	-	-	2.55	0.01
ci	<0.01	<0.01	0.03	-	99.07	-	<0.01	0.01	0.89	<0.01
cii	<0.01	<0.01	<0.01	-	97.52	-	-	<0.01	2.47	<0.01
ciii	<0.01	<0.01	<0.01	-	97.86	-	-	<0.01	2.13	<0.01
civ	<0.01	<0.01	<0.01	-	99.21	-	-	-	0.79	<0.01
cv	<0.01	<0.01	<0.01	-	97.43	-	-	-	2.57	0.01
cvi	<0.01	0.01	-	-	91.57	-	-	-	8.41	<0.01
cvii	0.01	0.01	-	-	88.43	-	-	<0.01	11.53	0.03
cviii	0.03	0.03	-	-	0.58	-	0.05	<0.01	99.14	0.18
ni	0.01	0.02	<0.01	-	75.19	0.01	-	0.04	24.68	0.06
nii	0.12	0.03	-	<0.01	92.22	<0.01	-	<0.01	7.6	0.03
niii	0.02	0.03	-	-	42.1	-	-	<0.01	57.8	0.05
niv	0.01	0.02	<0.01	-	53.38	-	-	<0.01	46.52	0.06
nv	0.01	0.01	-	-	78.98	-	<0.01	0.02	20.97	0.01
nvi	0.01	0.01	-	-	61.9	-	-	<0.01	38.05	0.03
nvii	<0.01	<0.01	-	-	99.1	-	-	<0.01	0.9	<0.01
nviii	<0.01	<0.01	<0.01	<0.01	97.19	<0.01	<0.01	<0.01	2.8	<0.01
nix	<0.01	<0.01	-	-	99.1	<0.01	-	0.02	0.88	<0.01
nx	<0.01	<0.01	-	<0.01	97	<0.01	0.01	0.01	2.98	<0.01

Supplementary Table S8. First four principal components from the unscaled viral transcript counts data in bees. Proportion of total variance explained is given in brackets.

	**$PC1_{bees}$ (95.0%)**	**$PC2_{bees}$ (5.0%)**	**$PC3_{bees}$ (<0.01%)**	**$PC4_{bees}$ (<0.01%)**
Aphid lethal paralysis virus	<0.0001	<0.0001	0.0012	-0.0066
Apis rhabdovirus 1	-0.0007	-0.0013	0.9956	0.0313
Apis rhabdovirus 2	<0.0001	<0.0001	0.0008	0.0047
Black queen cell virus	0.0235	0.6226	0.0202	-0.7739
Bundaberg bee virus 2	<0.0001	<0.0001	-0.0011	-0.0006
Chronic bee paralysis virus	<0.0001	<0.0001	-0.0038	-0.0034
Deformed wing virus	0.9994	-0.0347	0.0006	0.0024
Drosophila subobscura Nora virus	<0.0001	<0.0001	-0.0011	0.0035
Hobart bee virus 1	<0.0001	<0.0001	-0.0015	-0.0042
Hubei picorna-like virus 15	<0.0001	<0.0001	0.0121	-0.0027
Kashmir bee virus	0.0005	0.016	-0.0308	0.0996
Lake Sinai virus 1	0.0005	-0.0004	0.0264	-0.1044
Lake Sinai virus 3	-0.0001	<0.0001	0.0792	-0.0177
Sacbrood virus	0.0256	0.7816	-0.0137	0.6145
Varroa destructor virus 2	<0.0001	-0.0001	0.0044	0.0341
Varroa destructor virus 5	<0.0001	<0.0001	0.0013	0.0063
Vespa velutina Moku virus	<0.0001	<0.0001	-0.001	-0.0031

Supplementary Table S9. First four principal components from the analysis on the unscaled viral transcript counts data in *V. destructor*. Proportion of total variance explained is given in brackets.

	**$PC1_{Varroa}$ (89.0%)**	**$PC2_{Varroa}$ (11.0%)**	**$PC3_{Varroa}$ (<0.01%)**	**$PC4_{Varroa}$ (<0.01%)**
Apis rhabdovirus 1	0.0001	-0.0007	-0.9343	-0.2452
Apis rhabdovirus 2	-0.0001	-0.0005	-0.2441	-0.0713
Black queen cell virus	<0.0001	<0.0001	0.0121	-0.077
Bundaberg bee virus 2	<0.0001	<0.0001	0.0001	-0.0003
Deformed wing virus	0.8832	-0.469	0.0004	0.0001
Hubei picorna-like virus 22	<0.0001	<0.0001	-0.0012	0.0186
Kashmir bee virus	-0.0001	-0.0002	0.0083	-0.1437
Sacbrood virus	-0.0001	-0.0003	0.0274	-0.2426
Varroa destructor virus 2	-0.469	-0.8832	0.0011	-0.0011
Varroa destructor virus 5	-0.0007	-0.0013	-0.258	0.9214

Supplementary References

1 Yue, C. & Genersch, E. RT-PCR analysis of Deformed wing virus in honeybees (*Apis mellifera*) and mites (*Varroa destructor*). *J. Gen. Virol.* **86**, 3419-3424, doi:10.1099/vir.0.81401-0 (2005).

2 Bradford, E. L., Christie, C. R., Campbell, E. M. & Bowman, A. S. A real-time PCR method for quantification of the total and major variant strains of the Deformed wing virus. *PLoS One* **12**, e0190017, doi:10.1371/journal.pone.0190017 (2017).

3 Ongus, J. R. *et al.* Complete sequence of a picorna-like virus of the genus Iflavirus replicating in the mite *Varroa destructor*. *J. Gen. Virol.* **85**, 3747-3755, doi:10.1099/vir.0.80470-0 (2004).

4 Herrero, S. *et al.* Identification of new viral variants specific to the honey bee mite *Varroa destructor*. *Exp. Appl. Acarol.* **79**, 157-168, doi:10.1007/s10493-019-00425-w (2019).

5 Singh, R. *et al.* RNA viruses in hymenopteran pollinators: evidence of inter-taxa virus transmission via pollen and potential impact on non-*Apis* hymenopteran species. *PLoS One* **5**, e14357, doi:10.1371/journal.pone.0014357 (2010).

6 Benjeddou, M., Leat, N., Allsopp, M. & Davison, S. Detection of acute bee paralysis virus and black queen cell virus from honeybees by reverse transcriptase pcr. *Appl. Environ. Microbiol.* **67**, 2384-2387, doi:10.1128/AEM.67.5.2384-2387.2001 (2001).

7 Peng, W. *et al.* Host range expansion of honey bee Black queen cell virus in the bumble bee, *Bombus huntii*. *Apidologie* **42**, 650-658, doi:10.1007/s13592-011-0061-5 (2011).

8 Palacios, G. *et al.* Genetic analysis of Israel acute paralysis virus: distinct clusters are circulating in the United States. *J. Virol.* **82**, 6209-6217, doi:10.1128/JVI.00251-08 (2008).
